# An Innovative Failure Criterion for Metal Cylindrical Shells under Explosive Loads

**DOI:** 10.3390/ma15134376

**Published:** 2022-06-21

**Authors:** Yan Li, Wen Wang, Zhanfeng Chen

**Affiliations:** 1 School of Mechanical Engineering, Hangzhou Dianzi University, Hangzhou 310018, China; liyan@wfust.edu.cn (Y.L.); wangwn@hdu.edu.cn (W.W.); 2 Facility Horticulture Laboratory of Universities in Shandong, Weifang University of Science and Technology, Weifang 262700, China; 3 Provincial Key Laboratory of Oil and Gas Storage and Transportation Safety in Shandong Province, China University of Petroleum (Huadong), Qingdao 266580, China

**Keywords:** failure criterion, metal cylindrical shells, failure pressure, explosive load

## Abstract

Metal cylindrical shells are widely used to store and transport highly hazardous chemicals. The impact resistance of metal cylindrical shells under an explosive load is a concern for researchers. In this paper, an innovative failure criterion considering the time effect is proposed for metal cylindrical shells under explosive loads. Firstly, based on the maximum shear stress criterion, an innovative failure criterion containing the time effect is provided. Then, a metal cylindrical shell model is established. Next, a failure pressure equation for metal shells under an explosive load is proposed based on the innovative failure criterion. Lastly, the proposed equation is verified by numerical simulation. The results indicate the failure pressure equation for a metal cylindrical shell under an explosive load uses the finite element method. Our research is of significance for fully understanding the failure mechanism of piping and pressure vessels under impact load.

## 1. Introduction

The failure of a metal structure is usually caused by various loads in the environment, among which, the explosive load is one of the most damaging. At present, the research on the dynamic behavior of materials is mostly concentrated on aviation and the military industry. Explosive vessels are typical pressure vessels subjected to explosive loads that carry potential risks; they are usually metal, cylindrical shells. Metal cylindrical shells are used to transport hazardous materials and for scientific experiments [1]. The explosion test is a consumable test with a high cost and high risk factor. Explosive containers in the process of use carry the possibility of causing serious injury if they fail. In order to ensure the safety performance of metal cylindrical shells in service, accurate calculation of the dynamic failure pressure during the explosion is a key issue.

At present, the research methods of the failure pressure of metal cylindrical shells mainly include experimental methods, finite element methods and theoretical analysis. Most researchers’ studies have focused on the failure pressure of metallic cylindrical shells under static loading. In theoretical analysis, thin-walled cylinder theory and thick-walled cylinder theory are commonly used to predict the burst pressure, combined with the existing static strength criteria. Failure pressure is closely related to dynamic failure criteria for metal cylindrical shells under explosive loads. In order to understand the dynamic failure criteria of metal cylindrical shells under explosive loads, we should know the static failure criteria. There are significant differences and similarities between static failure criteria and dynamic ones. There has been a lot of research on static failure pressure and static failure criteria [2]. As early as the 1950s, Cooper [3] began to analyze the failure pressures of metal cylindrical shells using the von Mises criterion. Later, scholars also provided a number of failure pressure equations based on different failure criteria for cylindrical metal shells [4]. Recently, Zhu and Leis [5] proposed the Zhu–Leis criterion for the failure pressure of metal cylindrical shells. Zhang et al. [6,7] proposed the mean yield criterion for the failure pressure of metal cylindrical shells. Yu et al. [8,9] proposed the double shear yield criterion and unified strength theory for the failure pressure of metal cylindrical shells. Chen et al. [10] proposed a new multi-parameter failure criterion and applied it to the failure strength assessment of hydrogen pipelines. There are also some industrial standards for the failure pressures of metal cylindrical shells, such as ASME B31G [11], API [12], Nadai [13], and the ISO 10400 [14]. Investigation found that the existing failure criteria are for the failure pressures of materials under static loads and do not consider the time effect and inertia effect of materials under the action of instantaneous explosive loads.

Few researchers have studied the failure pressures of metal cylindrical shells under explosive loads. The failure criteria and failure pressures of metal cylindrical shells under impact loads are poorly studied, despite there being much research on the dynamic responses of metal cylindrical shells under explosive loads. Cheng et al. [15] investigated the dynamic responses and structural losses of cylindrical composite structures under repeated blast loading. V Hadavi et al. [16] proposed a theoretical method for calculating the maximum radial deflection of cylindrical shells under blast loading. Robert A [17] quantified the stress induced by shock waves within the pipeline using finite element methods. However, the failure pressure under dynamic explosive loading is slightly understudied. Finite element analysis is one of the most widely used methods for simulating the failure of pipes under static and dynamic loads, and also for validating the results of theoretical analyses [18,19]. Xue et al. [20] investigated the applicability of finite element analysis methods for predicting rupture pressure under dynamic and static internal pressure, and the results indicated that both dynamic and static finite element methods can predict burst pressure. Cheng et al. [21,22] proposed an empirical formula based on experimental data for determining the short-term burst pressures of metal cylinders under short-term dynamic loads. Wei et al. [23] took the gas cylinder steel HP295 as their research object and proposed a modified Barlow formula for the failure pressure of the gas cylinder. Chen et al. [24,25] proposed a failure pressure model for metal cylindrical shells subjected to explosive loads. However, the above studies did not theoretically analyze the failure behavior of metal cylindrical vessels under instantaneous blast loading, nor is the stress–strain behavior of the inner wall of a pipe under dynamic loading sufficiently understood. Failure criteria of metal cylindrical shells under explosive loads are critical for dynamic safety assessments of metal cylindrical shells. Therefore, it is necessary to establish an innovative failure criterion considering the time effect to further research the failure behavior of metal cylindrical shells under explosive loads.

As indicated above, despite the surging interest in the failure pressures of metal cylindrical shells under explosive loads, only limited attention has been given to a failure criterion that is applicable to the explosive loads. Consider that under explosive loads, the dynamic ultimate bearing capacity of a material is different from the static one, and the dynamic ultimate bearing capacity is related to time. To better understand the failure pressures of metal cylindrical shells under explosive loads, we propose an innovative failure criterion considering the time effect in this paper. Firstly, taking metal cylindrical shells as the research object, the explosive load is simplified according to the load characteristics. Secondly, based on the stress function method, we obtain the analytical solution for the metal cylindrical shell. Next, the failure pressure equation is developed in conjunction with the proposed failure criterion. In the end, the accuracy of the new failure pressure equation is verified by FEM.

## 2. An Innovative Failure Criterion

The mechanical behavior of a material subjected to explosive loads is significantly different from that subjected to static loads. The relationship between material failure pressure and strain rate is complicated. Under the action of explosive loads, the failure of metal materials is related to the time effect, inertia effect, and damping effect. Baker et al. [26] and Hampton et al. [27] reviewed and summarized the data of some carbon steels, and obtained a functional relationship between the strain rate and dynamic ultimate strength.

(1) $$\sigma_{u}^{d}=\sigma_{u}\left[1.1+0.1\log(\dot{\varepsilon }•s)\right]$$

where is $\sigma_{u}^{d}$ the dynamic ultimate strength of the materials; $\sigma_{u}$ the ultimate strength of the materials; $\dot{\varepsilon }$ is the strain rate, which can be calculated by the ratio of strain rate to time; *s* is the unit of time.

The Tresca criterion considers that the material will yield or fail if the maximum stress reaches a certain value, which can be expressed as:

(2) $$\sigma_{T}=\max(\left|\sigma_{1}-\sigma_{2}\right|,\left|\sigma_{2}-\sigma_{3}\right|,\left|\sigma_{3}-\sigma_{1}\right|)$$

When $\sigma_{1}>\sigma_{2}>\sigma_{3}$, the Tresca criterion can be simplified as:

(3) $$\sigma_{T}=\sigma_{1}-\sigma_{3}$$

where $\sigma_{T}$ is the Tresca effective stress. $\sigma_{1},\sigma_{2},\sigma_{3}$ are the first principal stress, the second principal stress, and the third principal stress, respectively.

Equation (3) shows that the intermediate principal stress does not affect the yield or failure of the material. Equation (3) cannot be directly used for metal cylinder shells under explosive load, because it does not take into account the time effect. The failure pressure of metal cylinder shells under an explosive load is time-dependent. A dynamic failure assessment can be performed only after the time effect is added to Equation (3). It should be clearly pointed out that adding the time effect into the Tresca criterion is the main innovation of this paper.

Previous studies have shown that the Tresca criterion is the lower limit of the predicted value when predicting the failure pressure of metal cylinder shells [5,28]. For the prediction of burst pressure under an explosive load, a conservative calculation is beneficial to reduce the failure rate of the vessel, so the relatively conservative predicted value is acceptable. To depict the failure criterion of materials under explosive loads, we introduce a coefficient *k* into Equation (3), which is from Equation (1). Additionally, the innovative failure criterion can be expressed as:

(4) $$\sigma_{T}^{d}=k\sigma_{T}^{}=\sigma_{1}^{’}-\sigma_{3}^{’}$$

where $k=1/1.1+0.1\log(\dot{\varepsilon }•s)$; $\sigma_{T}^{d}$ is Tresca effective stress; $\sigma_{1}^{’},\sigma_{3}^{’}$ are the first principal stress and third principal stress.

Equation (4) is the innovative failure criterion we propose in this paper. This criterion takes into account the strain rate and is quite different from the static damage criterion. The strain rate is a time-dependent parameter. Therefore, Equation (4) is an innovative failure criterion considering the time effect. As the innovative failure criterion was mainly proposed by Li Yan and Chen Zhan-Feng, it can also be called the Li–Chen criterion. The innovative failure criterion is a failure criterion transformed from a static failure criterion. Based on the innovative failure criterion, the failure pressure of a metal cylindrical shell under an explosive load can be transformed into a static load problem to be solved. This is similar to D’Alembert’s principle. The innovative failure criterion provides a new idea for further study of the dynamic failure behavior of materials.

## 3. Mechanical Model of Metal Cylinder Shells

In this section, the innovative failure criterion is first used in the failure pressure analysis of metal cylinder shells under explosive loads. The metal cylinder shell is used as the research object to study the burst pressure under internal explosive loads. When the burst load acts on the inner wall of the metal cylinder, the action time is brief, and the strain rate of the metal cylinder material is high. The classical static failure criterion cannot be applied during the explosion.

### 3.1. Geometric Model

The metal cylinder shell is simplified as a thick-walled, cylindrical container. To simplify the calculation, we performed the following assumptions:

1. The wall of a metal cylindrical shell is made of isotropic elastoplastic metal;
2. The impact of the metal fragments generated on the shell is not considered when the blast occurs;
3. The gas–solid interactive effect during the explosion is not considered;
4. No theoretical analysis of the elastic-plastic response of the cylindrical shell is performed;
5. We neglect the effect of axial load on the breaking pressure;
6. Explosions occur in routine environments.

### 3.2. Stress Boundary Condition

**Figure 1:**
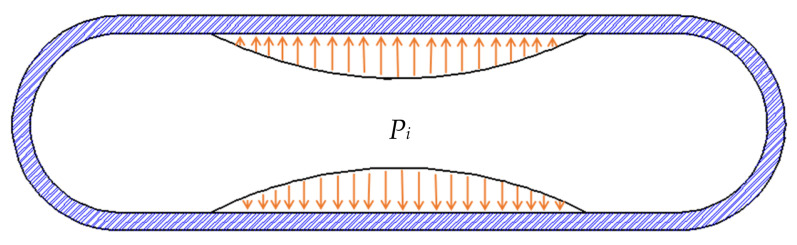
Geometric model of a metal cylindrical shell subjected to an internal explosive load.

It is not difficult to understand that the blast load distribution within the cylindrical shell is not uniform when the explosion occurs. In general, the load near the explosion point is greater than the load at the principle explosion point. Far from the explosion point, the load is low. Based on the above analysis, assuming that the explosion point is located in the center of the metal cylinder, the burst load on the plane can be simplified as two symmetrical parabolic loads. The parabolic load acts on the inner wall of the metal cylindrical shell, as shown in Figure 1; $P_{i}$ is the internal explosive load in the metal cylindrical shell. Ma et al. [1] made similar assumptions when analyzing the damage to metallic cylindrical shells under blast loading based on the finite element method. The difference is that the geometric model proposed in this paper can be used for general theoretical analysis and has significant theoretical significance.

## 4. Failure Pressure of Metal Cylindrical Shells

When the explosion occurs inside the metal cylindrical shell, the strong pulse generated acts on the inner wall of the metal cylindrical shell. The pulses are bounced back, become smaller, and eventually disappear. The first pulse is the most powerful, the most harmful, which is the focus of our research. Plenty of researchers are interested in the dynamic response. Unfortunately, only a few scholars have paid attention to the failure pressures and failure criteria of metal cylindrical shells. Presently, what is known is still not enough for scholars to study the failure mechanisms of metal cylindrical shells under explosive loads. The existing failure criterion makes it difficult to obtain the failure pressures of metal cylindrical shells under explosive loads.

**Figure 2:**
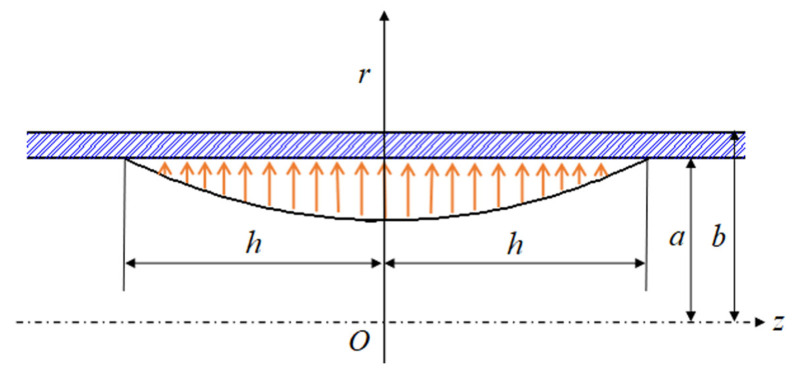
The mechanical model of a metal cylindrical shell under the first pulse. *a* is the internal radius of the metal cylindrical shell, *b* is the external radius of the metal cylindrical shell, *h* is the axial displacement of the explosive load from the maximum value to zero, *r* is radial displacement of the metal cylindrical shell.

In this section, we try to study the failure pressure of a metal cylindrical shell under an explosive load. During the explosion, the impact energy will gradually dissipate, and the intensity of the pulse will gradually decrease. The first pulse is the most destructive. Therefore, the first pulse is the key to determining the failure pressure. The first pulse closest to the explosion point has the most energy, whereas the pulse energy further from the explosion point is decreased. In this paper, the failure pressure of a metal cylindrical shell is determined by a failure pressure equation, regardless of the impacts of various solid fragments on the inside wall and gas–solid coupling. Considering the symmetry of the metal cylindrical shell, strain, stress, and displacement are also symmetrical under ideal conditions, as indicated in Figure 2.

### 4.1. Stress Function

Metal cylindrical shells are constructed from thick-walled, cylindrical structures with closed ends. According to the previous assumptions, as shown in Figure 2, it can be known that in the shell, all stress boundary conditions, the structure, and constraints are symmetrical around the *z*-axis. The relationship between stress and deformation of the cylindrical pressure vessel can be thought of as being determined by *r* and *z*, whereas angle θ has no bearing on it at all.

The stress function for the metal cylindrical shell conforms to the biharmonic equation [29]:

(5) $$\left(\frac{\partial^{2}}{\partial r^{2}}+\frac{1}{r}\frac{\partial }{\partial r}+\frac{\partial^{2}}{\partial z^{2}}\right)\left(\frac{\partial^{2}\phi }{\partial r^{2}}+\frac{1}{r}\frac{\partial \phi }{\partial r}+\frac{\partial^{2}\phi }{\partial z^{2}}\right)=0$$

The stress function of the metal cylindrical shell under a parabolic load can be expressed as:

(6) $$\begin{array}{cc} \varphi = & \gamma_{1}\left(8z^{5}-15zr^{4}\right)+\gamma_{2}\left(8z^{5}-40z^{3}r^{2}+15zr^{4}\right)+\gamma_{3}\left(8z^{4}-3r^{4}\right)+\\ & \gamma_{4}\left(2z^{4}-3r^{2}z^{2}\right)+\gamma_{5}z\log(r)+\gamma_{6}z^{3}+\gamma_{7}r^{2}z+\gamma_{8}zr^{2}\log(r)+\\ & \gamma_{9}z^{3}\log(r)+\gamma_{10}r^{2}\log(r)+\gamma_{11}z^{2}\log(r) \end{array}$$

where $\gamma_{1}$ to $\gamma_{11}$ are unknown coefficients that can be determined by the stress boundary condition; φ is the stress function of the metal cylindrical shell under a parabolic load.

### 4.2. Stress Component

In an axisymmetric problem, the stress components depend on *r* and *z* instead of θ. Following the stress function method of elastic theory, the stress component of the metal cylindrical shell can be expressed as [30]:

(7) $$\left\{\begin{array}{l} \sigma_{r}=\frac{\partial }{\partial z}\left(\mu \nabla^{2}\varphi -\frac{\partial^{2}\varphi }{\partial r^{2}}\right)\\ \sigma_{\theta }=\frac{\partial }{\partial z}\left(\mu \nabla^{2}\varphi -\frac{1}{r}\frac{\partial \varphi }{\partial r}\right)\\ \sigma_{z}=\frac{\partial }{\partial z}\left((2-\mu )\nabla^{2}\varphi -\frac{\partial^{2}\varphi }{\partial z^{2}}\right)\\ \tau_{rz}=\frac{\partial }{\partial r}\left((1-\mu )\nabla^{2}\varphi -\frac{\partial^{2}\varphi }{\partial z^{2}}\right) \end{array}\right.$$

where $\sigma_{r},\sigma_{{}_{\theta }},\sigma_{z},\tau_{rz}$ are the radial, hoop, axial, and shear stress, respectively. μ is the Poisson ratio, φ is the stress function of the cylindrical shell under a parabolic load, *r* is the radial displacement, and *z* is the axial displacement.

### 4.3. Boundary Conditions

The geometric representation of a metal cylindrical shell is shown in Figure 2. The inner radius of the cylindrical shell is parameter *a*, and the outer radius is parameter *b*. Axis *z* is the symmetric axis of the geometric model. As illustrated in Figure 2, a cylindrical coordinate system is created along the *z*-axis, and the parabolic pressure ranges from *h* to *–h* according to the actual impact range of the first pulse. Based on the mathematical assumption that *h* = *b*, the stress boundary conditions for the metal cylindrical shells can be determined as follows:

When r=a,

(8) $$\left\{\begin{array}{l} \sigma_{r}=a_{1}z^{2}+a_{2}z+a_{3}\\ \tau_{rz}=0 \end{array}\right.$$

where $a_{1},a_{2},a_{3}$ are the coefficients of the explosive load. When r=b, it can be obtained from Equation (8):

(9) $$\left\{\begin{array}{l} \sigma_{r}=0\\ \tau_{rz}=0 \end{array}\right.$$

When z=±h, it can be obtained from Equation (9):

(10) $$\int_{a}^{b}r\sigma_{z}d_{r}=0$$

By substituting Equation (6) into (7), we obtain the stress component. The expression is as follows:

(11) $$\left\{\begin{array}{l} \sigma_{r}=\left(480\gamma_{1}\mu +240\gamma_{2}+\frac{3\gamma_{9}}{r^{2}}\right)z^{2}+\left[192\gamma_{3}\mu +12\gamma_{4}(1+2\mu )+\frac{2\gamma_{11}}{r^{2}}\right]z+\left[180\gamma_{1}(3-4\mu )-180\gamma_{2}\right]r^{2}\\ \;\;\;\;\;\;\;+\left[6\gamma_{9}\mu +\gamma_{8}(4\mu -2)\right]\log r+\frac{\gamma_{5}}{r^{2}}-3\gamma_{8}+6\gamma_{6}\mu +4\gamma_{8}\mu +\gamma_{7}(4\mu -2)\\ \sigma_{\theta }=\left(480\gamma_{1}\mu +240\gamma_{2}-\frac{3\gamma_{9}}{r^{2}}\right)z^{2}+\left[192\gamma_{3}\mu +12\gamma_{4}(1+2\mu )-\frac{2\gamma_{11}}{r^{2}}\right]z+\left[60\gamma_{1}(1-4\mu )-60\gamma_{2}\right]r^{2}\\ \;\;\;\;\;\;\;+\left[6\gamma_{9}\mu +\gamma_{8}(4\mu -2)\right]\log r-\frac{\gamma_{5}}{r^{2}}-\gamma_{8}+6\gamma_{6}\mu +4\gamma_{8}\mu +2\gamma_{7}(2\mu -1)\\ \sigma_{z}=480\left[\gamma_{1}(1-\mu )-\gamma_{2}\right]z^{2}-24\left[8\gamma_{3}(\mu -1)+\gamma_{4}\mu \right]z+240\left[\gamma_{1}(\mu -2)+\gamma_{2}\right]r^{2}\\ \;\;\;\;\;\;\;+2\left[2\gamma_{8}(\mu -2)+3\gamma_{9}(1-\mu )\right]\log r+4\gamma_{7}(2-\mu )+4\gamma_{8}(2-\mu )+6\gamma_{6}(1-\mu )\\ \tau_{rz}=\left[\left(480\gamma_{1}(\mu -1)+480\gamma_{2}\right)r+\frac{4\gamma_{8}(1-\mu )-6\gamma_{9}\mu }{r}\right]z+\left[96\gamma_{3}(\mu -1)+12\gamma_{4}\mu \right]r\\ \;\;\;\;\;\;\;+\frac{4\gamma_{10}(1-\mu )-2\gamma_{11}\mu }{r} \end{array}\right.$$

where μ is Poisson’s ratio.

By substituting Equation (11) into Equations (8)–(10), the coefficients $\gamma_{1}$ to $\gamma_{11}$ can be obtained:

(12) $$\left\{\begin{array}{l} \gamma_{1}=\frac{a^{2}a_{1}}{240\left(a^{2}-b^{2}\right)(1+\mu )}\\ \gamma_{2}=\frac{a^{2}a_{1}(1-\mu )}{240\left(a^{2}-b^{2}\right)(1+\mu )}\\ \gamma_{3}=\frac{a^{2}a_{2}\mu }{96\left(a^{2}-b^{2}\right)(1+\mu )}\\ \gamma_{4}=\frac{a^{2}a_{2}(1-\mu )}{12\left(a^{2}-b^{2}\right)(1+\mu )}\\ \gamma_{5}=-\frac{a^{2}b^{2}\left[a^{2}a_{1}\mu +4a_{2}(1+\mu )\right]}{4\left(a^{2}-b^{2}\right)(1+\mu )}+\frac{4\mu a^{4}a_{1}b^{4}(\log b-\log a)}{4{\left(a^{2}-b^{2}\right)}^{2}(1-\mu )}\\ \gamma_{6}=-\frac{a^{2}\left(4a_{2}(\mu -2)+a^{2}a_{1}(\mu -1)+a_{1}b^{2}(1+3\mu )\right)}{12\left(a^{2}-b^{2}\right)(1+\mu )}+\frac{a_{1}a^{2}b^{2}\left(a^{2}\log a-b^{2}\log b\right)}{3{\left(a^{2}-b^{2}\right)}^{2}}\\ \gamma_{7}=\frac{a^{2}\left[\left(4a_{2}{\left(\mu -1\right)}^{2}+a_{1}\mu \left(a^{2}(\mu -1)-b^{2}(3+\mu )\right)\right)\right]}{8\left(a^{2}-b^{2}\right)\left(\mu^{2}-1\right)}+\frac{\mu a_{1}a^{2}b^{2}\left(b^{2}\log b-a^{2}\log a\right)}{2{\left(a^{2}-b^{2}\right)}^{2}(\mu -1)}\\ \gamma_{8}=\frac{a^{2}a_{1}b^{2}\mu }{2\left(a^{2}-b^{2}\right)(\mu -1)}\\ \gamma_{9}=-\frac{a^{2}a_{1}b^{2}}{3\left(a^{2}-b^{2}\right)}\\ \gamma_{10}=\frac{a^{2}a_{2}b^{2}\mu }{4\left(a^{2}-b^{2}\right)(\mu -1)}\\ \gamma_{11}=-\frac{a^{2}a_{2}b^{2}}{2\left(a^{2}-b^{2}\right)} \end{array}\right.$$

By substituting Equation (12) into Equation (11), the axial stress $\sigma_{z}$, the radial stress $\sigma_{r}$, the hoop stress $\sigma_{\theta }$, and the shear stress $\tau_{rz}$ of a metal cylindrical shell under the first pulse can be obtained:

(13) $$\left\{\begin{array}{l} \sigma_{r}=\frac{a^{2}a_{1}\left(b^{2}-r^{2}\right)}{\left(b^{2}-a^{2}\right)r^{2}}z^{2}+\frac{a^{2}a_{2}\left(b^{2}-r^{2}\right)}{\left(b^{2}-a^{2}\right)r^{2}}+\frac{a^{2}a_{1}\mu \left(b^{2}-r^{2}\right)\left(a^{2}-r^{2}\right)}{4\left(b^{2}-a^{2}\right)r^{2}\left(\mu +1\right)}-\\ \;\;\;\;\;\;\;\;\frac{a^{2}a_{1}b^{2}\mu }{{\left(b^{2}-a^{2}\right)}^{2}r^{2}\left(\mu -1\right)}\left[b^{2}\left(a^{2}-r^{2}\right)\log b+a^{2}\left(b^{2}-r^{2}\right)\log a-r^{2}\left(b^{2}-a^{2}\right)\log r\right]\\ \sigma_{\theta }=-\frac{a^{2}a_{1}\left(b^{2}+r^{2}\right)z^{2}}{\left(b^{2}-a^{2}\right)r^{2}}-\frac{a^{2}a_{2}\left(b^{2}+r^{2}\right)}{\left(b^{2}-a^{2}\right)r^{2}}-\frac{a^{4}a_{1}b^{2}\mu }{4\left(b^{2}-a^{2}\right)r^{2}\left(\mu +1\right)}+\\ \;\;\;\;\;\;\;\;\frac{a^{2}a_{1}\mu \left(a^{2}\left(\mu -1\right)+3r^{2}\left(1-\mu \right)+b^{2}\left(3+5\mu \right)\right)}{4\left(b^{2}-a^{2}\right)\left(1-\mu^{2}\right)}+\\ \;\;\;\;\;\;\;\;\frac{a^{2}a_{1}b^{2}\mu }{{\left(b^{2}-a^{2}\right)}^{2}r^{2}\left(\mu -1\right)}\left[b^{2}\left(r^{2}+a^{2}\right)\log b-a^{2}\left(r^{2}+b^{2}\right)\log a+r^{2}\left(a^{2}-b^{2}\right)\log r\right]\\ \sigma_{z}=\frac{2a_{1}a^{2}b^{2}\left(b^{2}\log b-a^{2}\log a\right)}{{\left(a^{2}-b^{2}\right)}^{2}\left(\mu -1\right)}+\\ \;\;\;\;\;\;\;\;\frac{a_{1}a^{2}\left[b^{2}\left(1+3\mu \right)+2r^{2}\left(1-\mu \right)+a^{2}\left(\mu -1\right)+4b^{2}\left(1+\mu \right)\log r\right]}{2\left(a^{2}-b^{2}\right)\left(\mu^{2}-1\right)}\\ \tau_{rz}=0 \end{array}\right.$$

### 4.4. Failure Pressure Equation

The failure pressure of a vessel under internal pressure usually refers to the maximum load carrying capacity under static or quasi-static loading. The static failure pressure can be obtained by hydrostatic experiment. In the experiment, the hydraulic loading rate must be very low, which can be regarded as a static or quasi-static process. The blast load is different from the static or quasi-static load in the experiment, which is a dynamic load. Depending on the strain rate of the material, a distinction can be made between static and dynamic loads. When the strain rate is less than $10^{-5}\;s^{-1}$, it is a static load. When the strain rate is between 10^−5^ and 10^−3^ s^−1^, it is a quasi-static load. When the strain rate is more than 10^−3^ s^−1^, it is a dynamic load [31]. The internal explosive load of the metal cylindrical shell is a characteristic dynamic load subject to strong time dependence. A number of equations have been proposed in the past for the prediction of the failure pressures for cylindrical vessels under static loads [32,33,34]. Since the explosive load is a time-dependent equation, these equations cannot be used for the prediction of the failure pressure of a metal cylindrical shell under an explosive load.

In this study, we assume that the first pulse is a parabolic impact load. It is evident that the maximum impact load of the parabolic load arises at *z* = 0. Consequently, the location of maximum stress is found in the positions of *z* = 0 and *r* = *a* in the cylindrical coordinate system. As *z* = 0 and *r = a* are substituted into Equation (13), the stresses of the metal cylindrical shell under explosive loads can be expressed as:

(14) $$\left\{\begin{array}{l} \sigma_{\theta }=-\frac{a_{3}\left(b^{2}+a^{2}\right)}{\left(b^{2}-a^{2}\right)}+\frac{a^{2}a_{1}\mu \left(b^{2}(1+3\mu )+a^{2}(1-\mu )\right)}{2\left(b^{2}-a^{2}\right)\left(1-\mu^{2}\right)}+\frac{2a^{2}a_{1}b^{4}\mu (\log b-\log a)}{{\left(b^{2}-a^{2}\right)}^{2}(\mu -1)}\\ \sigma_{z}=\frac{a^{2}a_{1}\left(b^{2}(1+3\mu )+a^{2}(1-\mu )\right)}{2\left(b^{2}-a^{2}\right)\left(1-\mu^{2}\right)}+\frac{2a^{2}a_{1}b^{4}(\log b-\log a)}{{\left(b^{2}-a^{2}\right)}^{2}(\mu -1)}\\ \sigma_{r}=a_{3} \end{array}\right.$$

where $\sigma_{\theta },\sigma_{z},\sigma_{r}$ corresponded to $\sigma_{1},\sigma_{2},\sigma_{3}$.

To simplify the expressions, we make the following assumption:

(15) $$\left\{\begin{array}{l} A=-\frac{\left(b^{2}+a^{2}\right)}{\left(b^{2}-a^{2}\right)}\\ B=\frac{a^{2}\mu \left(b^{2}(1+3\mu )+a^{2}(1-\mu )\right)}{2\left(b^{2}-a^{2}\right)\left(1-\mu^{2}\right)}\\ C=\frac{2a^{2}b^{4}\mu (\log b-\log a)}{{\left(b^{2}-a^{2}\right)}^{2}(\mu -1)} \end{array}\right.$$

As a result, the first principal stress can be described as follows:

(16) $$\sigma_{1}=Aa_{3}+(B+C)a_{1}$$

As for the third principal stress, it can be expressed as follows:

(17) $$\sigma_{3}=a_{3}$$

By substituting Equations (16) and (17) into Equation (4), we can obtain the following expression:

(18) $$Aa_{3}+(B+C)a_{1}-a_{3}=\sigma_{u}$$

By substituting z=b and $a_{2}=0$ into Equation (8):

(19) $$a_{1}b^{2}+a_{3}=0$$

By solving Equations (18) and (19), we can determine *a*_1_ and *a*_3_.

(20) $$\left\{\begin{array}{l} a_{1}=-\frac{\sigma_{u}}{k(1+Ab^{2}-B-C)}\\ a_{3}=\frac{b^{2}\sigma_{u}}{k(1+Ab^{2}-B-C)} \end{array}\right.$$

Obviously, $a_{3}$ is the maximum parabolic internal pressure. Based on the innovative failure criterion, the failure pressure equation of the metal cylindrical shell can be expressed as:

(21) $$P_{b}^{d}=\frac{b^{2}\sigma_{u}}{k(1+Ab^{2}-B-C)}$$

where $k=\frac{1}{1.1+0.1\log(\dot{\varepsilon }\cdot s)}$.

## 5. Verification of Accuracy by FEM

As the deformation process needs to consider the inertia effect and strain rate effect of the structure, the stress state of the implosion condition and its analysis method are fundamentally different from those for a static load [35]. FEM is a good tool for determining static and dynamic failure pressures in pressure vessels and pipes. To verify the accuracy of the new failure pressure equation and criterion, we analyzed a metal cylinder shell using LS-DYNA software under dynamic explosion conditions. In this section, the accuracy of Equation (21) is examined based on finite element simulation results in reference [22].

**Figure 3:**
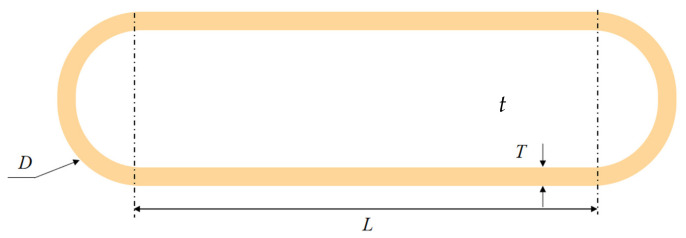
Shape and dimensions of the metal cylindrical shell.

The geometric model of the FEA is shown in Figure 3. The metal cylindrical shell is complete and defect-free. In Figure 3, *t* is the wall thickness of the metal cylindrical shell, *D* is the outer diameter of the metal cylindrical shell, and *L* is the length of the metal cylindrical shell (excluding the closed-end).

**Figure 4:**
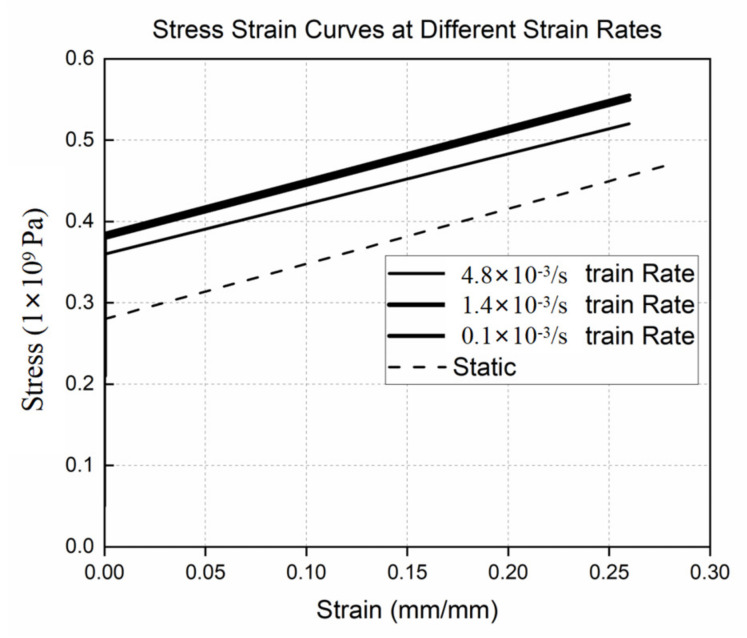
Stress–strain curve of ASTMA-106B steel.

**Figure 5:**
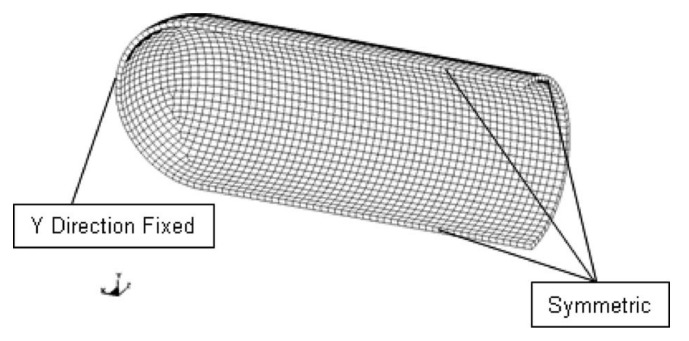
Boundary conditions [21].

**Figure 6:**
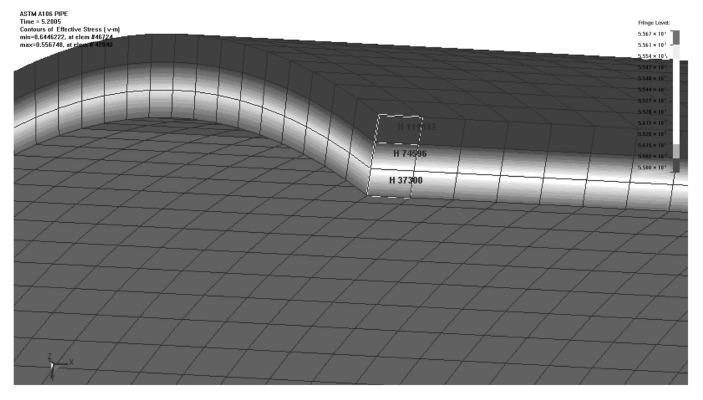
The von Mises stress distribution at 5.2005 ms [21].

The finite element analysis was performed on ASTMA-106B steel [27]. This material is primarily used for seamless steel pipes. A constitutive relationship for ASTM A-106B is depicted in Figure 4 at different strain rates. The failure criterion was the maximum plastic strain under different strain rates in the finite element analysis. After the plastic strain reached a maximum value for a particular strain rate in FEA simulation, the element was removed from the calculation. By examining the output of the FEA model, we can determine the failure pressure and burst time. Due to the boundary condition, loads, and symmetry of the geometry, a quarter model was employed in FEA. The boundary conditions are shown in Figure 5. It was fixed along the y-axis at the end of the cap, and gravity acted in the negative y-axis direction. Rotation was not restricted. Figure 6 shows the stress distribution in the cylindrical shell. The results of the finite element analysis are listed in Table 1.

According to the literature [21,36], LS-DYNA is used to simulate the dynamic failure of metal cylindrical shells. During the explosion, the impact load on the inner wall can generate several periodic pulses. The duration of the pulse is actually half of the pulse period. The pulse duration $t_{d}$ is from 3.9 to 390 ms. The pulse duration and the burst time are different in finite element analysis. The results of finite element analysis can be used to determine the burst time $t_{b}$ in Equation (21). The strain ratio is $\dot{\varepsilon }=\varepsilon /t_{b}$. Table 1 lists the burst time $t_{b}$ and pulse duration. ASTM A-160B steel has a maximum strain of 0.262. Thus, it is possible to obtain the strain rate $\dot{\varepsilon }$ by Equation. The tensile strength of ASTM A-160B steel in static tensile state is 413.69 MPa. Thus, the dynamic failure pressure $P_{b}^{d}$ can be calculated using Equation (21) based on the data above. The calculation results are presented in Table 1.

**Figure 7:**
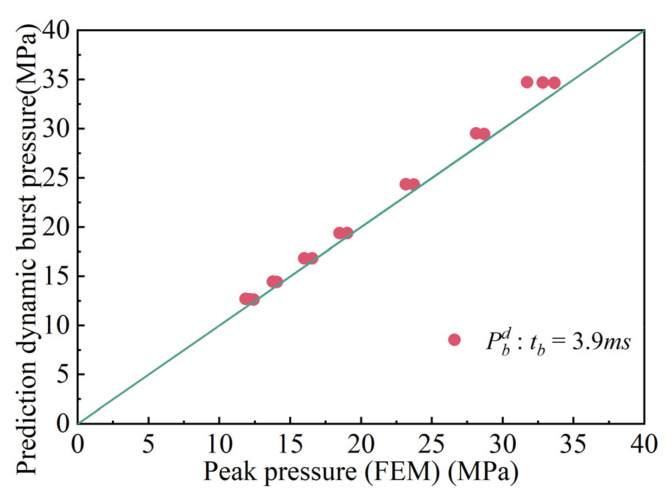
Comparison results with pulse duration $t_{d}=3.9\mathrm{ms}$.

**Figure 8:**
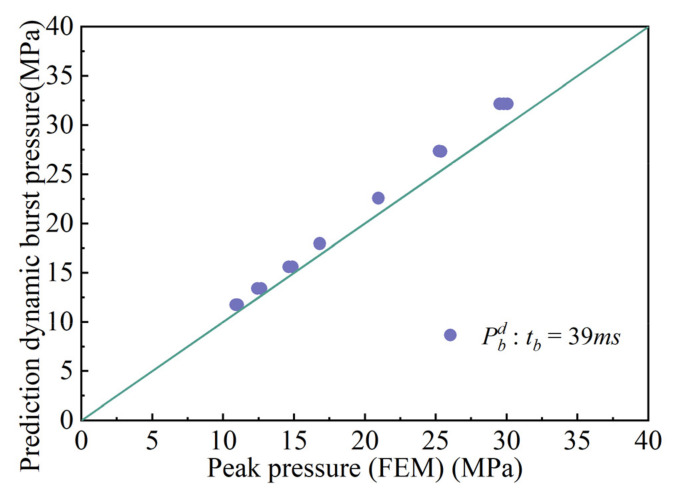
Comparison results with pulse duration $t_{d}=39\mathrm{ms}$.

**Figure 9:**
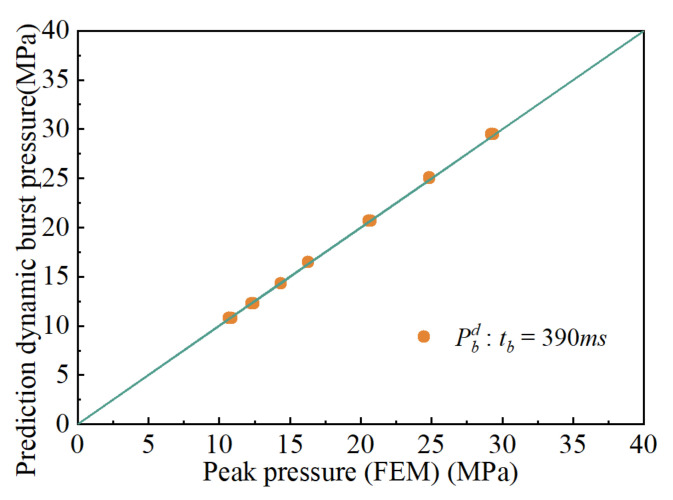
Comparison results obtained when $t_{d}=390\mathrm{ms}$.

Comparisons between FEA and the calculation results are shown in Figure 7, Figure 8 and Figure 9. Figure 7 demonstrates the comparison between the calculated results and the finite element results at *t_d_* = 3.9 ms. The maximum relative error in the comparison results is 9.46%, and the minimum relative error is 1.65%. The majority of the error ranges fall between 0% and 10%. Figure 8 shows the comparison of the calculated results and those obtained from finite elements at time *t_d_* = 39 ms. The maximum error is 9%, the minimum error is 4.9%, and the majority of errors are between 0% and 9%. Figure 9 shows the comparison between the calculated results and the finite element results at *t_d_* = 390 ms. The minimum error is 0.14%, and the maximum error is 1.19%. Most of the errors are less than 1%. Through comparison, it was found that when *t_d_* = 390 ms, the calculation results are the closest to the finite element analysis result, and the errors are the smallest.

This study aimed to establish an innovative failure criterion for dynamic strength analysis. Unlike earlier studies that simplified the explosive load within a metal cylindrical shell to a uniform load, in this study, the explosive load inside the metal cylindrical shell was simplified to a parabolic load. The analytical solution of the metal cylindrical shell was obtained. Validation of the new failure pressure equation with different pulse duration times was carried out by comparing our calculations with finite element analysis results. The results indicated that the new failure pressure equation can predict the failure pressure accurately. The innovative failure criterion suggested in this paper can be used to analyze the mechanical behavior of materials under explosive loads. The innovative failure criterion and failure pressure equation of metal cylindrical shells under explosive loads can be regarded as a reference for the design of cylindrical vessels.

## 6. Conclusions

In this paper, the dynamic failure problem of a metal cylindrical shell under an explosive load was studied. Firstly, a dynamic strength criterion containing time was proposed based on the Tresca strength criterion. Secondly, in order to analyze the stress components when the metal cylindrical shell is subjected to the explosive load, the explosive load was simplified to a symmetric parabolic load in the plane, and the stress components of the explosive container under the burst load were obtained. Thirdly, a predicted equation of dynamic failure pressure was obtained based on the innovative failure criterion. Finally, the proposed dynamic failure pressure equation was validated by FEM. The results suggest that the values calculated by the failure pressure equation based on the innovative failure criteria are reasonably close to the values calculated by the FEM. Prediction accuracy is greatest when the pulse duration is 390 milliseconds. The minimum error was 0.14%, and the maximum error was 1.19%. The prediction accuracy increases with an increase in pulse duration. It is the subject of our next study to identify the specific reasons for this. This study provides a prediction method for dynamic failure pressure and also provides a theoretical reference for the failure of metal cylindrical shells under dynamic loading.

## Figures and Tables

**Table 1:** Finite element analysis results and theoretically calculated $P_{b}^{d}$.

No.	*D* (mm)	*T* (mm)	$t_{d}\;(\mathrm{ms})$	Peak Pressure (MPa)	$t_{b}\;(\mathrm{ms})$	$P_{b}^{d}\;(\mathrm{MPa})$
1	355.6	4.191	3.9	12.4106	5.011	12.6449
	355.6	4.191	39	11.0317	40.461	11.743
	355.6	4.191	390	10.7007	391.456	10.7631
2	355.6	4.7752	3.9	14.0654	5.236	14.4245
	355.6	4.7752	39	12.6864	40.064	13.4207
	355.6	4.7752	390	12.4106	398.019	12.2881
3	355.6	5.5372	3.9	16.5475	4.916	16.8208
	355.6	5.5372	39	14.8928	39.626	15.6229
	355.6	5.5372	390	14.3412	387.939	14.3133
4	355.6	6.35	3.9	19.0296	4.9046	19.363
	355.6	6.35	39	16.8233	40.522	17.9678
	355.6	6.35	390	16.2717	395.383	16.4627
5	355.6	7.9248	3.9	23.7181	5.002	24.3216
	355.6	7.9248	39	20.9602	41.239	22.5697
	355.6	7.9248	390	20.6844	388.973	20.7061
6	355.6	9.525	3.9	28.6824	4.965	29.4512
	355.6	9.525	39	25.3729	41.170	27.3246
	355.6	9.525	390	24.8213	393.792	25.0444
7	355.6	11.1252	3.9	33.6466	4.968	34.6446
	355.6	11.1252	39	30.0613	40.438	32.1649
	355.6	11.1252	390	29.3718	391.481	29.48
8	266.7	3.14452	3.9	12.1348	4.724	12.6756
	266.7	3.14452	39	10.8938	40.635	11.746
	266.7	3.14452	390	10.7559	388.724	10.7706
9	266.7	3.5814	3.9	13.7896	4.790	14.4684
	266.7	3.5814	39	12.4106	40.876	13.4108
	266.7	3.5814	390	12.2727	388.998	12.2994
10	266.7	4.1529	3.9	15.9959	4.825	16.8315
	266.7	4.1529	39	14.6170	39.905	15.6188
	266.7	4.1529	390	14.3412	387.140	14.3145
11	266.7	4.7625	3.9	18.4781	4.738	19.3858
	266.7	4.7625	39	16.8233	39.863	17.9786
	266.7	4.7625	390	16.2717	393.439	16.466
12	266.7	5.9436	3.9	23.1665	4.738	24.3667
	266.7	5.9436	39	20.9602	40.297	22.5889
	266.7	5.9436	390	20.5465	390.747	20.7023
13	266.7	7.1501	3.9	28.1308	4.657	29.5429
	266.7	7.1501	39	25.2350	40.769	27.3598
	266.7	7.1501	390	24.8213	392.350	25.0813
14	266.7	8.3439	3.9	32.8192	4.748	34.6982
	266.7	8.3439	39	29.7855	40.384	32.1665
	266.7	8.3439	390	29.2340	401.020	29.4516
15	177.8	5.5626	3.9	31.7161	4.670	34.7178
	177.8	5.5626	39	29.5097	40.396	32.1661
	177.8	5.5626	390	29.2340	392.020	29.4784
16	177.8	2.0955	3.9	11.8591	4.426	12.715
	177.8	2.0955	39	10.8938	39.783	11.7503
	177.8	2.0955	390	10.8938	387.844	10.7671
